# Does the Relief of Glucose Toxicity Act As a Mediator in Proliferative Actions of Vanadium on Pancreatic Islet Beta Cells in Streptozocin Diabetic Rats?

**DOI:** 10.6091/ibj.1329.2014

**Published:** 2014-07

**Authors:** Leila Pirmoradi, Mohammad Taghi Mohammadi, Akbar Safaei, Fakhardin Mesbah, Gholam Abbas Dehghani

**Affiliations:** 1 *Dept. of Physiology, *; 2 *Dept. of Pathology, *; 3 *Dept. of Anatomy, Nemazi Hospital, Shiraz University of Medical Sciences, Shiraz, Iran*

**Keywords:** Vanadium, Rats, Diabetes, Protection, Beta cells

## Abstract

**Background:** Data shows vanadium protects pancreatic beta cells (BC) from diabetic animals. Whether this effect is direct or through the relief of glucose toxicity is not clear. This study evaluated the potential effect of oral vanadyl sulfate (vanadium) on glycemic status and pancreatic BC of normal and diabetic rats. **Methods:** Rats were divided into five groups of normal and diabetic. Diabetes was induced with streptozocin (40 mg/kg, i.v.). Normal rats used water (CN) or vanadium (1 mg/ml VOSO_4_, VTN). Diabetic rats used water (CD), water plus daily neutral protamine Hagedorn insulin injection (80 U/kg, ITD) or vanadium (VTD). Blood samples were taken for blood glucose (BG, mg/dL) and insulin (ng/dL) measurements. After two months, the pancreata of sacrificed rats were prepared for islet staining. **Results:** Pre-treated normal BG was 88 ± 2, and diabetic BG was 395 ± 9. The final BG in CD, VTD, and ITD was 509 ± 22, 138 ± 14, and 141 ± 14, respectively. Insulin in VTN (0.75 ± 0.01) and VTD (0.78 ± 0.01) was similar, higher than CD (0.51 ± 0.07) but lower than CN (2.51 ± 0.02). VTN islets compared to CN had larger size and denser central core insulin immunoreactivity with plentiful BC. CD and ITD islets were atrophied and had scattered insulin immunoreactivity spots and low BC mass. VTD islets were almost similar to CN. **Conclusion:** Besides insulin-like activity, vanadium protected pancreatic islet BC, and the relief of glucose toxicity happening with vanadium had a little role in this action.

## INTRODUCTION

Diabetes mellitus, a state of chronic hyper-glycemia, is characterized by increased insulin resistance of peripheral tissues (type 2 diabetes) or reduced pancreatic beta cell (BC) mass (type 1 diabetes). The increased insulin resistance in type 2 diabetes initially is compensated by stimulation of pancreatic islets to raise insulin secretion, but the final outcome is exhaustion and degeneration of BC [1-4].

Streptozocin (STZ) is a toxic glucose analogue that irreversibly destroys pancreatic BC and induces type 1 diabetes in laboratory animals, and it is generally used to study the *in vivo* effects of antidiabetic drugs as well as vanadium compounds [5]. Even though the elimination of hyperglycemia after induction of diabetes prevents the diabetogenic effects of STZ [6], long-term hyperglycemia (glucose toxicity) deteriorates diabetes and irreversibly damages the viable BC survived from STZ [7]. 

Hyperglycemia, hypoinsulinemia, and the trophy of pancreatic islets and the depletion of insulin contents are the obvious signs of STZ diabetes [7-10]. Studies carried out in STZ diabetic animals have demonstrated that oral vanadium dramatically improves peripheral tissue responsiveness to circulating insulin (insulin) and promotes a stable normoglycemia even after withdrawal [11, 12]. Regardless of decreased insulin level in normal and diabetic rats, insulin-like activity of vanadium enhanced glucose utilization and perhaps with the relief of glucose toxicity prevented further destruction of pancreatic BC [13-15]. 

The beneficial insulin-like activity and the proliferative effects of oral vanadium on reliving hyperglycemia and the pancreatic BC of STZ-diabetes rats are well documented [8, 12, 16, 17]. In spite of the presence of euglycemia in normal rats, vanadium also distended pancreatic islets by proliferation of their BC [14, 16]. Furthermore, vanadium, in addition to the elimination of hyperglycemia, prevented the atrophy of pancreatic islets of partially diabetic rats by protecting their BC [14, 16, 18]. Although long-term hyperglycemia (glucose toxicity) deteriorates diabetes and irreversibly damages the viable BC that survived from STZ [7], the elimination of hyperglycemia with insulin injection if happens shortly after induction of diabetes prevents the diabetogenic effects of STZ [6]. It is well documented chronic hyperglycemia is responsible for oxidative stress leading to BC dysfunction and loss [19]. Consequently, the elimination of hyperglycemia which happens with vanadium treatment [13, 15, 16, 20] may help the pancreatic islets of diabetic rats to re-establish their functions. Therefore, the present study was designed to examine the influence of removal of glucose toxicity in the proliferation of BC and islet insulin stores reported in vanadium-treated diabetic rats. This idea was tested with daily injection of neutral protamine Hagedorn (NPH) insulin in chronic hyperglycemia in STZ diabetic.

## MATERIALS AND METHODS

Male normal Sprague Dawley rats (200-250 g) were obtained from Central Animal House Facility of Shiraz University of Medical Sciences (Shiraz, Iran). All protocols of the study were approved by the Institutional Animal Ethics Committee of the University, which follows NIH guidelines for care and use of animals (NIH publication No. 85-23, revised in 1996). The animals were housed in standard cages in a room with controlled temperature (22-24°C), humidity (40-60%), and light period (07.00-19.00), while having access to food (rat food, Parsdam, Tehran, Iran) and fluid *ad libitum*. 

***Fluid solutions. ***The drinking water (base solution) contained 3 g/L NaCl in distilled water to overcome the problems of natriuresis which occurs in diabetic rats [12]. Vanadyl solution contained 1 mg/ml vanadyl sulfate (VOSO_4_ + 5H_2_O, Merck, Germany) in base solution. The solutions were prepared freshly every 3-5 days and stored in a dark cold room (4^o^C) until use.

***Blood samples.*** The animals were lightly anesthetized with ether, and a blood sample (500 µL) was collected from the tip of snipped tail. Two µL was used to measure blood glucose (BG) using Glucose Monitoring System (Glucocard 01-Mini, Japan), the rest was centrifuged (12,000 ×g), and its serum was stored in a freezer (-70^°^C) for the assessment of plasma insulin.

***Routine measurements*** *. *The drinking fluid was measured daily for the first two weeks and every other day thereafter. Bodyweight was determined weekly. BG was measured routinely and the serum insulin was measured at times mentioned in the results section.

***Induction of diabetes and maintenance of the animals. ***Rats were made diabetic with a single i.v. injection of freshly prepared solution of STZ (40 mg/kg in normal saline) through lateral tail vein. Diabetes was confirmed after 48-72 h of STZ injection by the presence of hyperglycemia (BG = 350-400 mg/dL), polydipsia (fluid intake ≥ 100 mL/day), and polyuria (wetting cages). Control normal animals received i.v. the same volume of normal saline [8]. 

***Experimental design.*** Normal and diabetic rats were randomly divided into 5 experimental groups: Control normal (CN, n = 12) received base solution as drinking water for 60 days. Vanadyl-treated normal rats (VTN, n = 12) received vanadyl solution as drinking water for 60 days. The concentration of vanadyl was low at first (0.1 mg/mL), within two weeks gradually increased to 1 mg/mL and consumed thereafter. Control diabetic rats (CD, n = 9) received base solution as drinking water for two months. Treatment of diabetic rats started 10 days after STZ injection. In vanadyl-treated diabetic rats (VTD, n = 11), fluid was switched to vanadyl solution. The concentration of vanadyl solution started from 0.05 mg/ml and with the reduction of water consumption, increased to 1 mg/mL. Insulin-treated diabetic (ITD, n = 10) received daily intraperitoneal injection of NPH insulin (80 U/kg, Exir, Iran) plus base solution. During the first week of insulin therapy, the required dose was about 100-120 U/kg. After this initial resistance, the dose was gradually decreased and BG was kept at the level of VTD with a stable dose of 80 U/kg.

***Preparation of the pancreas*** ***. ***After two-month experiments, animals were killed under deep anesthesia (ketamine/xylazine 70/10 mg/kg). The whole pancreas was gently dissected out and prepared for staining. The prepared pancreatic tissues were randomly assigned for hematoxilen & eosin (H&E), toluidine blue, or immunohistochemical staining. The samples were obtained from the same region of the pancreas to have better judgments about the changes that may occur in islets and their BC.

***Histological observations.*** Five rats of each group were used for histological staining. The sections were stained with H&E or toluidine blue staining using the routine protocols, and the rest were prepared for immunohistochemistry.

***Immunohistochemical staining of pancreatic islets. ***Pancreas was fixed in 10% neutral buffered formalin (pH 7.4) at room temperature for 48 h. After fixation and dehydration, in a graded series of ethanol, paraffin blocks were cut into 4-micron-thick sections. The sections were deparafﬁnized with xylene, rehydrated in a descending graded series of ethanol and then rinsed with 0.02 M PBS. Three-five-tissue sections of each pancreas were prepared for BC immune-fluorescence staining. The activity of endogenous peroxidases was blocked by submerging paraffin-embedded tissues in methanol containing 3% hydrogen peroxide. The use of non-immune serum helped to eliminate non-specific background colors. Primary anti-insulin clone antibody (Sigma I-2018) was used to detect insulin containing BC. The sections were successively incubated with biotinylated antibody for 20 min and rinsed with PBS. Histostain-Plus kit (Zymed Code 85- 9943) was used according to the methods described by other investigators [16, 21]. The use of aminoethyl carbazole created an intense red deposit around the antigen/antibody enzyme complex. The sections were arbitrarily coded and examined using light microscopy by two independent experts. 

***Statistical analysis*** *.* Data are presented as mean ± SE. Wilcoxon-Newman and Mann-witney tests were used to obtain the statistical differences among the means of intergroup and between the groups for BG and circulating insulin. Repeated measurement test was used for the comparison of body weight and fluid consumption. Values were considered statistically significant when *P*<0.05.

## RESULTS

***Body weight and water intake.*** Changes in body weight and daily fluid consumption occurred during two-month experiments are presented in Table 1. A steady-state increase in body weight was observed during the first 10 days in CN and VTN, whereas during experiment, this increase was continued in CN and ITD. Also, no changes in body weight were observed in VTN, CD, and VTD. Table 1 also presents daily fluid consumption. The mean average water intake of normal rats before treatments was 35 ± 1 mL/day and did not change over time in CN. Vanadium treatment in VTN significantly reduced water intake (20 ± 2 mL/day). In CD during the first 10 days of diabetes, water intake was increased (128 ± 6 mL/day, *P*<0.001). This increase was plateaued after three to four weeks and stayed at 200 ± 9 mL/day (*P*<0.001). The elimination of polydipsia happened in VTD (11 ± 1 mL/day) or ITD over time, and the decrease was significant at the end of the experiment. However, the reduced water consumption by ITD (73 ± 5 mL/day) was always higher than CN (*P*<0.05). 

***Blood glucose and insulin levels. ***Fasting BG and circulating insulin levels (insulin) in normal and diabetic rats are presented in Table 2. Before the start of the experiments, the respective mean levels of BG and insulin were 88 ± 2 mg/dL and 2.51 ± 0.02 ng/dL respectively. These values did not statistically change over time in CN. However, normoglycemia persisted in VTN (BG = 81 ± 5 mg/dL) and CN, but 10 days after STZ injection, severe hyperglycemia (BG = 395 ± 9 mg/dL) with low level of insulin (0.75 ± 0.01 ng/dL) was observed in diabetic rats. Hyperglycemia was worsened in CD during two months (BG = 509 ± 22 mg/dL), and insulin was further decreased and reached to 0.51 ± 0.07 ng/dL (*P*<0.001). With a 53% increase in insulin (0.78 ± 0.01 ng/dL, *P*<0.001), high BG was gradually decreased in VTD and over time at BG = 138 ± 14 mg/dL. In ITD, the chosen dose of NPH insulin (80 U/kg/day) lowered BG close to BG in VTD (141 ± 14 mg/dL). Even though there was a significant increase in the level of insulin in VTD (0.78 ± 0.01ng/dL), but still it was significantly lower than CN (*P*<0.015). While BG in VTD did not significantly change for one week after vanadium withdrawal, in ITD, the re-occurrence of hyperglycemia (BG>400 mg//dL) was observed 48 h after cessation of NPH insulin.

**Table 1:** Body weight (BW, g) and fluid consumption (F, mL/day) of the rats before and 10, 40, and 70 days after STZ injection or vanadium treatment

** Days** **Groups**	**Before**			**10**			**40**			**70**			***P*** ** value**	
	**BW**	F		**BW**	**F**		**BW**	**F**		**BW**	**F**		**BW**	F
CN (n = 12)	214 ± 3	30 ± 1		227 ± 4	33 ± 1		245 ± 3	40 ± 1		282 ± 4	34 ± 1		<0.001	0.001
														
VTN (n = 12)	217 ± 3	35 ± 1		239 ± 9	35 ± 4		219 ± 9	21 ± 1		213 ± 6	20 ± 2		0.033	0.006
														
CD (n = 9)	227 ± 6	38 ± 1		226 ± 6	136 ± 6		220 ± 8	187 ± 7		220 ± 7	200 ± 9		0.016	<0.001
														
VTD (n = 11)	227 ± 5	37 ± 2		226 ± 5	115 ± 4		210 ± 6	36 ± 3		204 ± 8	11 ± 1		0.001	<0.001
														
ITD (n = 10)	223 ± 7	40 ± 2		218 ± 7	133 ± 6		250 ± 20	75 ± 10		283 ± 20	73 ± 5		0.042	<0.001
														
*P* value	0.336	0.009		0.185	<0.001		0.018	<0.001		<0.001	<0.001			

Data are as mean ± SE. CN, control normal; VTN, vanadyl-treated normal; CD, control diabetic; VTD, vanadyl-treated diabetic; ITD, insulin-treated diabetic

**Table 2:** Blood glucose (BG, mg/dL) and insulin concentrations (ng/dL) before, 10, 30, and 70 days of the treatment

** Days** **Groups**	**Before**			**10**		**30**			**70**			***P*** ** value**	
	**BW**	Insulin		**BG**		**BG**	**Insulin**		**BG**	**Insulin**		**BG**	Insulin
CN (n = 12)	87 ± 3	2.27 ± 0.02		86 ± 3		89 ± 5	2.35 ± 0.01		89 ± 3	2.45 ± 0.02		0.571	0.586
													
VTN (n = 12)	89 ± 3	2.87 ± 0.04		84 ± 6		85 ± 7	NM		81 ± 5	0.75 ± 0.01		0.083	0.005
													
CD (n = 9)	86 ± 4	2.59 ± 0.03		365 ± 16		377 ± 14	NM		509 ± 22	0.51 ± 0.07		<0.001	0.006
													
VTD (n = 11)	89 ± 4	2.42 ± 0.02		377 ± 19		277 ± 9	0.65 ± 0.01		138 ± 14	0.78 ± 0.01		<0.001	0.012
													
ITD (n = 10)	88 ± 3	2.52 ± 0.01		356 ± 13		256 ± 24	NM		141 ± 14	NM		<0.001	
													
*P* value	0.863	0.429		<001		<001	0.031		<0.001	0.015			

Data are as mean ± SE. CN, control normal; VTN, vanadyl-treated normal; CD, control diabetic; VTD, vanadyl-treated diabetic; ITD, insulin-treated diabetic; NM, not measured

**Fig. 1:**
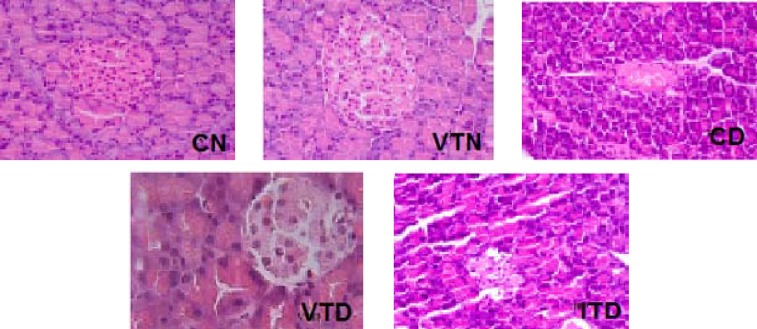
The histological photographs of hematoxilen & eosin staining of pancreatic tissues of normal and experimental rats (more details are given in the Materials and Methods section). The islets were distinct in CN and VTN groups. Islets of VTN were larger in size and increased beta cell numbers and mass in comparison to CN. In CD and ITD, the islets were atrophied and reduced in size and number, their beta cells were degenerated. In comparison with CD, islets in VTD were larger in size; close to normal islets of CN group (magnification 400×). CN, control normal; VTN, vanadyl-treated normal; CD, control diabetic; VTD, vanadyl-treated diabetic; ITD, insulin-treated diabetic

**Fig. 2.**
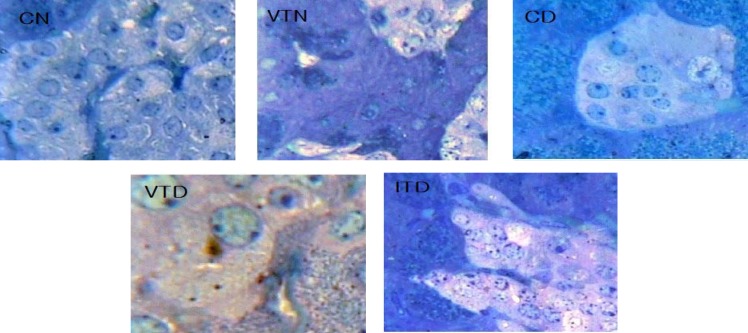
The histological photographs of toluidine blue staining of pancreatic tissues of normal and experimental rats (more details are described under Figure 1 and Materials and Methods section). In CN and VTN groups, islets were distinct. Islets of VTN were larger in size with profound beta cell number and mass. In CD and ITD, the islets were small with reduced beta cell number and mass. In comparison with CD, islets in VTD group were somewhat larger in size and their beta cell structure and numbers were near normal rats of CN group (magnification 400×). CN, control normal; VTN, vanadyl-treated normal; CD, control diabetic; VTD, vanadyl-treated diabetic; ITD, insulin-treated diabetic

***Light microscopy of pancreatic islets and their beta cells.*** The typical H & E and toluidine blue staining results of the pancreas obtained upon histological examinations are presented in Figures 1 and 2. The changes happened in other groups were compared with pancreatic histology of CN (Figs. 1 and 2, CN). The pancreas in VTN was grossly more spread over small intestine, islets were larger in size (Fig. 1, VTN), and BC situated centrally and were more abundant (Fig. 2, VTN). In contrast, in STZ untreated diabetic rats with severely damaged pancreas (Fig. 1, dispersed (Fig. 2, CD). Vanadium in diabetic rats (VTD) increased BC mass (Fig. 1, VTD), and with the expansion of islet areas, it partially repaired the damaged islets (Fig. 2, VTD). Even though hyperglycemia with NPH insulin had some protective actions on islets in ITD, islet areas and BC mass were still smaller than VTD (Figs.1 and 2, ITD).

**Fig. 3:**
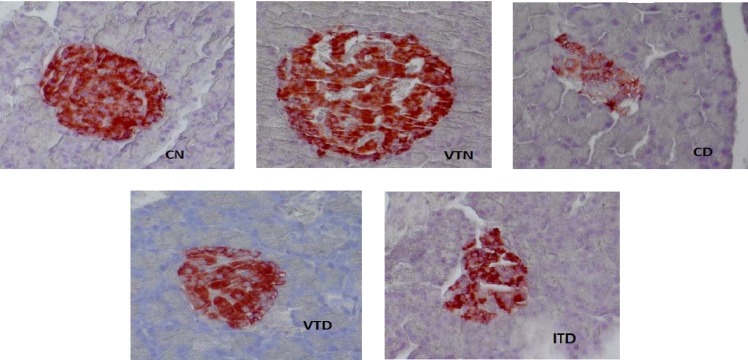
The immunohistochemical staining of the pancreatic islets of normal and experimental groups. Diabetes was induced with a single i.v. injection of STZ (40 mg/kg). CN and diabetic CD rats only received base solution (see the text). The treatments of the diabetic rats (VTD) with vanadium or NPH insulin (ITD) were started 10 days after the STZ injection. VTN rats also received oral vanadium for two months as described in Materials and Methods section. Comparisons are made between pancreatic islets of the five groups: CN, VTN, CD, VTD, and ITD. The typical distributions of the beta cells with moderate immunoreactivity are well seen in the CN and VTN. A weak immunoreaction was seen in few beta cells of the CD and ITD. Insulin immunoreactivity was highly increased in beta cells of VTN compared to CN, and VTD compared to CD or ITD (magnification 400 ×). CN, control normal; VTN, vanadyl-treated normal; CD, control diabetic; VTD, vanadyl-treated diabetic; ITD, insulin-treated diabetic

***Immunohistochemistry of pancreatic islet beta cells.*** Immunohistochemical staining of the pancreatic islets are presented in Figure 3. The pancreas in CN possessed normal islets with central clusters of BC (Fig. 3, CN). In VTN, the structure of islets was the same as CN, but insulin immunoreactivity of central core islets was increased (Fig. 3, VTN). Insulin immunoreactivity spots in CD was scattered, islets were atrophied and BC mass was reduced (Fig. 3, CD). In VTD in comparison to CD or ITD, insulin immunoreactivity spots were condensed centrally, islet areas were expanded, and BC mass was increased. However, there was a close similarity between islet areas and dispersed insulin immunoreactivity of ITD and CD. 

## DISCUSSION

The typical daily water intake, body weight gain, glycemic status, and pancreatic islet structures of normal and diabetic rats presented here are used to compare the influences of vanadium treatments on normal and diabetic rats. High BG, polydipsia, polyuria (wetting cages), and reduced insulin are clear signs of diabetes induced by STZ. The existence of atrophied islets, reduced BC mass, and dispersed small insulin immunoreactivity spots indicated that damages were permanent. This result agrees with the previous investigations [12, 16, 22].

The rate of weight gain was steady and positive in CN. However, the observed weight loss in CD, shortly after STZ injection, was conceivably linked with the impaired carbohydrate metabolism as a source of energy, or defects that happened in controlling energy homeostasis at the level of central nervous system [23]. Insulin therapy in ITD improved glycemic profile and reversed weight loss to a steady positive weight gain eventually reaching to CN. The initial suppression and the significant reduction in growth weight, seen in VTN and VTD, more likely were due restriction of food untake or anorexigenic stimulation of vanadium [24]. 

Glucose toxicity, which happens during chronic hyperglycemia, is responsible for BC dysfunction and loss in diabetes [19]. In this situation, the relief of glucose toxicity which happens during long-term vanadium consumption in diabetic rats may protect the viable BC and restored pancreatic islet functions [13, 15, 16, 20]. In the present study, we intended to eliminate chronic hyperglycemia in STZ diabetic rats with daily injection of NPH insulin, and see if the relief of glucose toxicity acts as a mediator in preserving pancreatic islet beta cells similar to what happens in vanadium-treated diabetic rats.

In this study, it was also observed that BG and daily water intake of diabetic rats were treated with vanadium or insulin, while the non-treated diabetic rats of CD stayed hyperglycemic with polydipsia during the experiment. Also, in VTD, vanadium gradually reduced both the elevated BG levels and daily water intake. The insulin-like activity of vanadium reduced BG by helping peripheral tissues to take up more glucose at a significantly low insulin [5, 8, 12, 16, 22]. The decreased water intake in ITD was one-third and well-coordinated with BG level, while this level was significantly higher than CN. The concept of deprived water intake seen in VTN or VTD might be related to the taste of vanadium solution, or its participation in the regulation of salt and water balance at the level of central nervous system [25]. 

As mentioned previously, the insulin-like activity of vanadium improves the sensitivity of peripheral tissues to insulin [12, 22, 26]. Therefore, regardless of islet expansion, increased BC mass [10, 14, 16], in VTN, less insulin (30% insulin in normal rats) was needed to maintain normoglycemia. However, the reduced BG in VTD in part was due to the increased insulin concentration (50% more than CD) and also insulin-like activity of vanadium [27, 28]. Similar to the previous studies, this study showed vanadium consumption in diabetic rats preserved BC and also islet atrophy [15, 16]. Previous studies revealed that the insulin-mimetic actions of vanadium in type 1 diabetic patients or insulin-dependent diabetic rats needed a minimum level of insulin to overcome hyperglycemia [12, 27, 29]. In this study, the insulin-mimetic action of vanadium in VTN was displayed after two weeks, whereas in VTD, the reduced BG began after four to six weeks after treatment. However, in both conditions, the improved carbohydrate metabolism and balanced glycemic status needed a minimum level of insulin [8, 10, 13, 15, 20]. 

Chronic hyperglycemia is toxic to BC. Some studies have stated that the elimination of chronic hyperglycemia with exogenous insulin, if started shortly after diabetes, suppresses the diabetogenic actions of STZ [6, 22]. Other studies performed in diabetic animals have shown hyperglycemia reduces the proliferative cell capacity [30]. In the pancreas, it is coupled with BC death and depletes pancreatic insulin stores which deteriorated glucose homeostasis [31]. Similar adverse effects of hyperglycemia may play a role in the development of type 2 diabetes in genetically susceptible individuals [4]. 

Even though the elimination of hyperglycemia shortly after induction of diabetes prevents the diabetogenic effects of STZ [6], long-term hyper-glycemia (glucose toxicity) deteriorates diabetes and irreversibly damages the viable beta cells that survived from STZ [7]. 

It is well documented that hyperglycemia is responsible for oxidative stress, which leads to beta cell dysfunction and loss in diabetes [19]. However, long-term vanadium consumption in conjunction with the elimination of hyperglycemia protected viable beta cells and restored pancreatic islet functions in diabetic rats [13, 15, 16, 20]. In the present study, we intended to eliminate chronic hyperglycemia in STZ diabetic rats, with daily injection of NPH insulin, and see if the relief of glucose toxicity acts as a mediator in preserving pancreatic islet beta cells similar to what happens in vanadium treated diabetic rats.

Many studies have demonstrated that vanadium in diabetic rats reduced BG levels, and if used for a longer period, it preserved the viable BC and by proliferation, it prevented islet atrophy and insulin depletion [10, 16, 22]. This study showed that the relief of glucose toxicity *per se*, which happened in VTD, did not act as a mediator to prevent BC destruction and islet atrophy. This event is because of islet atrophy, reduced BC mass as well as low, scattered and reduced insulin immunoreactivity spots, seen in CD, were still present in ITD. 

The result of this study indicated that the mechanisms of insulin-like activity of vanadium improved glucose homeostasis in STZ-diabetic rats. Furthermore, the insulinotropic activity of vanadium stimulated BC proliferation and replaced the damaged insulin secretory cells, and the relief of glucose toxicity happened in vanadium- or insulin-treated diabetic rats had a minimal role in this accomplishment.
